# A consensus approach toward the standardization of spinal stiffness measurement using a loaded rolling wheel device: results of a Delphi study

**DOI:** 10.1186/s12891-021-04313-6

**Published:** 2021-05-13

**Authors:** Maliheh Hadizadeh, Greg Kawchuk, Simon French

**Affiliations:** 1 Department of Physical Therapy, Faculty of Rehabilitation Medicine, University of Alberta, 3-48 Corbett Hall, Edmonton, AB T6G 2G4 Canada; 2 Department of Physical Therapy, Faculty of Rehabilitation Medicine, University of Alberta, 3-44 Corbett Hall, Edmonton, AB T6G 2G4 Canada; 3 Department of Chiropractic, Macquarie University, Sydney, New South Wales Australia

**Keywords:** Spinal stiffness, Mechanical instruments, VerteTrack, Spinal pain, Delphi

## Abstract

**Background:**

Spinal stiffness assessment has the potential to become an important clinical measure. Various spinal stiffness-testing devices are available to help researchers objectively evaluate the spine and patient complaints. One of these is VerteTrack, a device capable of measuring posteroanterior displacement values over an entire spinal region. This study aimed to develop a best-practice protocol for evaluating spinal stiffness in human participants using VerteTrack.

**Methods:**

Twenty-five individuals with research experience in measuring spinal stiffness, or who were trained in spinal stiffness measurement using the VerteTrack device, were invited to participate in this 3-Round Delphi study. Answers to open-ended questions in Round 1 were thematically analyzed and translated into statements about VerteTrack operation for spinal stiffness measurements. Participants then rated their level of agreement with these statements using a 5-point Likert scale in Rounds 2 and 3. A descriptive statistical analysis was performed. Consensus was achieved when at least 70% of the participants either strongly agreed, agreed, (or strongly disagreed, disagreed) to include a statement in the final protocol.

**Results:**

Twenty participants completed Round 1 (80%). All these participants completed Rounds 2 and 3. In total, the pre-defined consensus threshold was reached for 67.2% (123/183) of statements after three rounds of surveys. From this, a best-practice protocol was created.

**Conclusions:**

Using a Delphi approach, a consensus-based protocol for measuring spinal stiffness using the VerteTrack was developed. This standard protocol will help to improve the accuracy, efficiency, and safety of spinal stiffness measurements, facilitate the training of new operators, increase consistency of these measurements in multicenter studies, and provide the synergy and potential for data comparison between spine studies internationally. Although specific to VerteTrack, the resulting standard protocol could be modified for use with other devices designed to collect spinal stiffness measures.

**Supplementary Information:**

The online version contains supplementary material available at 10.1186/s12891-021-04313-6.

## Background

Low back pain (LBP) is the most burdensome of musculoskeletal conditions globally affecting ~ 7.5% of the world’s population (~ 577 million people) [1]. For up to 90% of people presenting with LBP, the specific cause of their pain cannot be clearly identified resulting in a label of non-specific LBP [2]. The current treatment of LBP mainly focuses on pain management while the causes of pain are rarely addressed*.* Quantitative assessments of the spine and patient complaints related to LBP may help with the identification of causes, improve the management of this condition, and reduce health care system costs.

Advances in science and technology over the past few decades have made several devices available to objectively assess clinical characteristics of patients including spinal stiffness. Stiffness is considered an important spinal biomechanical measure and has long been recognized by both patients and clinicians as one of the characteristic features of the back [3]. Therefore, stiffness has been widely used in the management of patients with back pain for diagnosis, prognosis, clinical decision-making, and the evaluation of manipulative techniques [4].

An increase or decrease in spinal stiffness has been found to be related to LBP. Specifically, previous studies have demonstrated that some patients with LBP have abnormal levels of spinal stiffness [5] and that these patients experience an immediate and sustained decrease in spinal stiffness for 1 week following spinal manipulative therapy [6, 7]. Moreover, researchers reported an increase in posteroanterior (PA) stiffness in participants with LBP compared to when participants had little or no pain, while asymptomatic controls showed insignificant changes in PA stiffness over time [8]. A reduction in stiffness has also been shown to be associated with self-reported measures of disability [6, 9]. These findings suggest that restoration of normal spinal stiffness and mobility plays an important role in some patients with LBP by improving spinal function and reducing pain although a casual relation between stiffness and these outcomes has not been confirmed. Therefore, further exploration of spinal stiffness assessment is warranted. While there are various spinal stiffness-testing devices available to objectively evaluate the spinal complaints [4, 5], there is no standard operating protocol for spinal stiffness measurement.

Having a standard data collection protocol for spinal stiffness assessment would facilitate comparison of devices and data between studies. Our research team developed a novel device, the VerteTrack, to improve on single-site spinal indentation by employing a loaded rolling wheel system. Several identical devices have been manufactured and are in use in multiple research centers over the past 6 years. In this Delphi study, our goal was to develop a best-practice protocol for evaluating spinal stiffness in human participants using VerteTrack, a spinal stiffness measurement device shown to be safe [10], reliable [11], and accurate [12].

## Methods

This study used a standard Delphi methodology to achieve consensus. The Delphi method is a reliable and structured method of obtaining a consensus of opinion from a group of experts or knowledgeable participants [13] in areas where existing research is limited. The Delphi method is particularly recommended for areas where controversy, debate, or a lack of clarity exist [14].

### Selection of participants

As our lab manufactured the device in question, we know of all the research centers that possess the device and all the staff who were trained on the device. We contacted these centers and asked them to provide us with an updated contact list of those who were trained/used the device since their initial training session. Thus, all individuals trained in VerteTrack methods and/or having previous experience using the VerteTrack device were invited to participate in the Delphi process (*n* = 25 individuals from 9 different institutions in 7 different countries). Potential participants were asked to participate in the study if they were willing to participate, have access to the internet over the course of the study, and were able to commit time to complete the surveys. Written consent was obtained from all participants after being informed about the project by adding a consent question to the start of Round 1.

### Delphi-survey procedure

The Delphi survey involved three sequential rounds of deidentified online questionnaires provided over 4 months (Sep-Dec 2020). Study data were collected and managed using REDCap [15] electronic data capture tools provided by the Women & Children’s Health Research Institute at the University of Alberta. We contacted the research centers that are equipped with the device and asked them to send us the email addresses of those who were trained or collected data using the device. E-mail addresses were then entered into the REDcap website. All potential participants were sent an invitation email to participate in the Delphi process containing a link to the online survey. Participants were requested to complete each questionnaire within 2 weeks. Two automated e-mail reminders per round were sent out to non-responders at 1 week and the day before the due date. If participants were not able to complete the questionnaires within the 2 weeks, they were provided with additional reminders and extra time to respond. Each survey took 20–30 min to complete. Participants were allowed to save their answers and return to complete the questionnaire over several sessions.

**Fig. 1:**
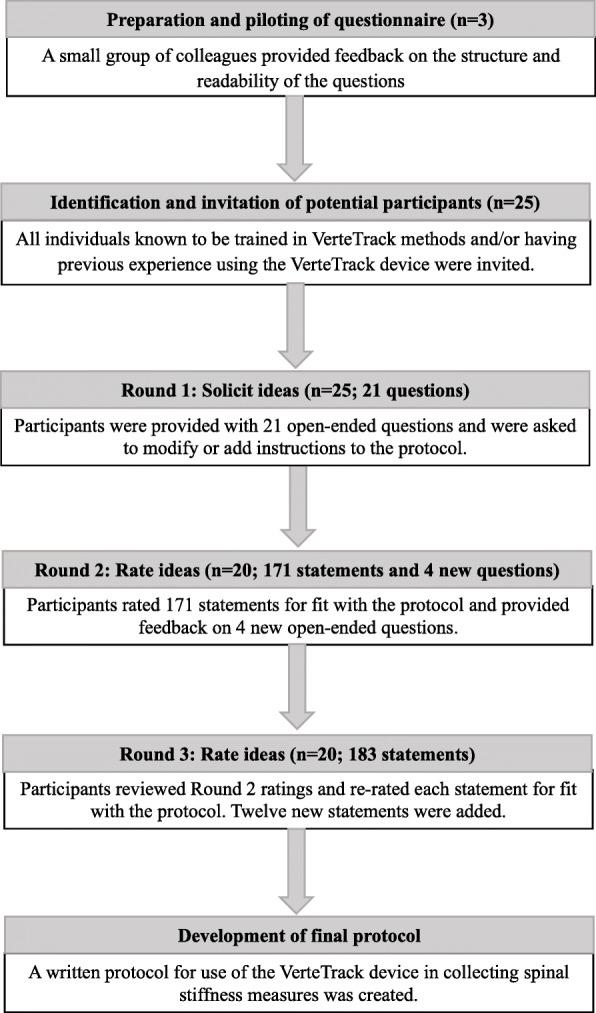
Stages of the Delphi technique to standardize spinal stiffness measurement using VerteTrack

Prior to the commencement of this study, consensus was defined when at least 70% of the participants in Rounds 2 and 3 either strongly agreed, agreed, (or strongly disagreed, disagreed) to include a statement in the final protocol. These levels of agreement have been considered appropriate in previous Delphi studies [13, 16–19]. Figure 1 summarizes the stages of the Delphi method in this study.

In order to improve the structure and readability of questions, the Round 1 questionnaire was first piloted with three colleagues. Based on their feedback, Round 1 questions were revised and finalized. MH and GNK designed the Round 1 of the survey. This round included questions regarding basic demographic information and 21 open-ended questions inquiring about participant recruitment for VerteTrack testing, device safety, instructions given to research participants, and technical issues. This round aimed to review the comprehensiveness and relevance of the items and provide suggestions for the eventual protocol. Items for Round 2 of the survey were generated by comments from the first round that suggested removing, aggregating, or retaining items from the first round.

Only those who completed round 1 were invited to participate in Round 2. In this round, each participant received a survey comprising 171 statements. The goal of this round was to reach consensus on a standard protocol. In Round 2, participants were asked to indicate their anonymous opinion by ranking statements along a five-point Likert scale for agreement (“strongly agree”, “agree”, “neither agree nor disagree”, “disagree”, “strongly disagree”). Additionally, a free-text comment section for each question was available for participants to express any further thoughts or opinions. Round 2 also included four new open-ended questions derived from Round 1. Participants were required to rate every single item to be able to move on with the questionnaire.

Round 3 of the study comprised the same list and grading scale as Round 2 with an additional graphical description of findings from the previous round. The graphic information identified the percentage of total respondents that selected each possible score for the given item in Round 2. The respondents, therefore, were given an opportunity to modify or confirm their answers after viewing the scoring results using the same Likert scale from the previous round. The revised and new statements proposed by participants were added in Round 3 yielding a total of 183 statements. Using the consensus results obtained from Round 3, the authors created a written protocol for use of the VerteTrack device in collecting spinal stiffness measures.

### Analysis

Deidentified data were analyzed by encoding participants with their survey ID numbers. Data from the REDCap tool was downloaded into a Microsoft Excel version 16.45 after each round. Descriptive statistics were used to describe the participants’ demographic characteristics. Responses to open-ended questions in the Round 1 and participants’ comments in Round 2 were thematically analyzed with MH and GNK discussing the qualitative responses. MH, GNK and SF met to discuss the items for the consensus statements in Rounds 2 and 3. The quantitative responses from the participants’ ratings in Rounds 2 and 3 were analyzed descriptively using medians, ranges, and percentages.

## Results

Of the 25 individuals invited to participate in this Delphi study, 20 participants completed Round 1 (80% response rate), 20/20 completed Round 2 (100.0% response rate), and 20/20 completed Round 3 (100.0% response rate). The reasons for 5/25 participants not responding to the initial invitation email were not identified. Table 1 presents the demographic characteristics of participants at baseline. Participants had different experiences working with the device that ranged from receiving training to performing measurements of spinal stiffness in a population of 180 patients with back pain.

**Table 1:** Baseline characteristics of Delphi participants (*n* = 20)

Baseline characteristics	Value^a^
Gender (% female)	35
Age (years)	32.5 ± 8.3
Years of clinical experience	5.6 ± 6.6
The country in which the measurement was performed (%).	
Australia	20
Canada	30
Denmark	15
France	5
Honk Kong	10
USA	20
Highest educational qualification (%)	
BSc	15
MSc	45
Ph.D.	35
D.C.	5
Occupation at the time of the study (%)	
Assistant professor	15
Senior lecturer	15
Post-doc fellow	5
Research coordinator	5
Research assistant	10
Student	20
Chiropractor	15
Physiotherapist	15
Primary discipline (%)	
Chiropractic	55
Physiotherapy	25
Other	20
The number of participants assessed using the VerteTrack device (min-max)	0–180

^a^ Values are mean ± SD unless otherwise indicated

In total, the pre-defined consensus threshold was reached for 67.2% (123/183) of statements after three rounds of surveys. Results from Round 3 were presented in Table 2. The number of consensus statements under each category was listed in Table 3. Items with 70% or more consensus from Round 3 were used to create the best practice protocol for the VerteTrack device (Additional file 1).

**Table 2:** Median value of Likert scale data and agreement level for all statements from Round 3

Domain	Consensus statement	Median (Range)	Percentage of respondents rating each statement		
			Agree (%)	Neutral (%)	Disagree (%)
Inclusion criteria	The ability to tolerate a load of at least 40 N.	1 (1–3)	95.0^a^	5.0	0.0
	BMI under 40 for ease of palpation.	1 (1–3)	85.0^a^	15.0	0.0
	18 years or older.	3 (1–4)	30.0	55.0	15.0
	Chronic back pain.	3 (1–5)	25.0	45.0	30.0
Exclusion criteria	Pregnancy.	1 (1–2)	100.0^a^	0.0	0.0
	Skin lesion, infection, or open wounds over the back region.	1 (1–1)	100.0^a^	0.0	0.0
	Unable to lie in the prone position (e.g., severe deformities to spine or limbs, static tremor, uncontrolled epilepsy).	1 (1–1)	100.0^a^	0.0	0.0
	Serious spinal pathology (e.g., spinal tumor, fracture, infectious disorder, osteoporosis, or other bone demineralizing condition).	1 (1–2)	100.0^a^	0.0	0.0
	Unable to maintain their breathing cycle in passive expiration (functional residual capacity) for at least 10 s.	1 (1–2)	100.0^a^	0.0	0.0
	Unable to follow instructions (e.g., those with dementia or children (age under 18) who may move during the test.	1 (1–3)	95.0^a^	0.5	0.0
	A head, neck, or thoracoabdominal surgery within the last 6 months.	1 (1–3)	90.0^a^	10.0	0.0
	Unstable spondylolisthesis.	1 (1–5)	85.0^a^	10.0	5.0
	Unstable and/or acute disc herniation or injury.	1.5 (1–4)	75.0^a^	20.0	5.0
	People who do not feel comfortable with the VerteTrack procedure.	1 (1–4)	75.0^a^	20.0	5.0
	Unstable heart condition.	2 (1–4)	70.0^a^	15.0	15.0
	Claustrophobia (a fear of being in closed or small spaces).	2 (1–5)	65.0	15.0	20.0
	Acute pain in the test area (depends on whether a participant can tolerate the loading and how long the aggravated pain will subside).	2 (1–5)	60.0	20.0	20.0
	Obesity using BMI (e.g., BMI > 30).	2 (1–4)	55.0	15.0	30.0
	Hyperalgesia (an abnormally increased sensitivity to pain).		55.0	25.0	20.0
	Obesity using waist circumference (e.g., waist circumference more than 35 in. in women).	2.5 (1–5)	50.0	15.0	35.0
	Previously sacrum trauma/sensitive sacrum.	3 (1–5)	45.0	25.0	30.0
	Spinal canal stenosis.	3 (1–5)	35.0	25.0	40.0
	Participants with exaggerated spinal curves e.g., thoracic hyper-kyphosis.	3 (1–5)	30.0	25.0	45.0
	People with asthma, colds, or breathing disorders.	4 (1–5)	25.0	20.0	55.0
	History of spine surgery (depends on whether a participant can tolerate the loading and how long the aggravated pain will subside).	3.5 (1–5)	25.0	25.0	50.0
	Scoliosis.	3.5 (1–5)	20.0	30.0	50.0
	Tenderness in the test area (depends on whether a participant can tolerate the loading and how long the aggravated pain will subside).	4 (1–5)	15.0	20.0	65.0
Pregnancy	A pregnant woman should not participate at any stage of pregnancy.	3 (1–5)	45.0	30.0	25.0
	From the first day of pregnancy to 3 months postpartum.	3 (1–5)	45.0	35.0	20.0
	Excluded from the second trimester.	3 (1–5)	40.0	35.0	25.0
	From confirmation of pregnancy till 6 weeks postpartum.	3 (1–5)	35.0	30.0	35.0
	From the first day of pregnancy till 1 month postpartum.	3 (1–5)	30.0	40.0	30.0
	From the first day of pregnancy to the day following the delivery.	3 (1–5)	25.0	35.0	40.0
	From confirmation of pregnancy to 12 months postpartum.	4 (1–5)	15.0	30.0	55.0
Participants’ familiarization procedures	Remind the participants once again some points to note e.g., hold breath during the measurement.	1 (1–2)	100.0^a^	0.0	0.0
	Make sure participants have understood the procedure and don’t have any questions.	1 (1–2)	100.0^a^	0.0	0.0
	Practice breathing protocol with the participant before beginning the measurements.	1 (1–2)	100.0^a^	0.0	0.0
	Some reassurance that while they may feel pressure on the spine, the device will not cause any harm.	1 (1–2)	100.0^a^	0.0	0.0
	Explain that there is an emergency stop.	1 (1–2)	100.0^a^	0.0	0.0
	Explain in detail the duration of the experiment and the set of data that needed to be collected.	1 (1–2)	100.0^a^	0.0	0.0
	Show participants the orientation video.	2 (1–4)	75.0^a^	20.0	5.0
	Show the device to the participant in person, pointing out the different parts and what their function is to help them further understand the process.	2 (1–4)	70.0^a^	25.0	5.0
	Orientation to the texture and feel of the rolling device.	2 (1–5)	65.0	30.0	5.0
	Allow an upper limit of 5 unloaded practice rounds and always note in the protocol how many practice rounds were completed.	2 (1–5)	65.0	20.0	15.0
	A sensory perception (load on hand).	3 (1–5)	35.0	35.0	30.0
	Watch someone else have the measures done (if this is not in the orientation video).	3 (2–5)	30.0	25.0	45.0
Instructions for participants before the assessment	You should wear clothes that can be moved to expose your waistline. A gown or shorts might be needed.	1 (1–2)	100.0^a^	0.0	0.0
	You have to empty your front and back pockets including coins, keys, cellphones.	1 (1–2)	100.0^a^	0.0	0.0
	You should remove your glasses.	1 (1–2)	100.0^a^	0.0	0.0
	You should go to the restroom before testing.	1 (1–2)	100.0^a^	0.0	0.0
	Explain and practice breathing protocol.	1 (1–2)	100.0^a^	0.0	0.0
	You should disrobe/change as necessary to expose the test area sufficiently.	1 (1–2)	100.0^a^	0.0	0.0
	You should wear comfortable clothing.	1 (1–2)	100.0^a^	0.0	0.0
	Explain some circumstances where the participant might want to press the emergency stop. E.g., if they have radicular pain, and they experience pain in their leg.	1 (1–4)	95.0^a^	0.0	5.0
	Explain how the device works to increase participant comfort.	1 (1–3)	95.0^a^	0.0	5.0
	Explain how to lay down.	2 (1–4)	80.0^a^	5.0	15.0
	Cell phones should be allowed to stay on for emergency calls etc. but the participant should be instructed that we don’t want them looking at their phones during the protocol.	2 (1–5)	65.0	15.0	20.0
Identifying the Spinous processes	Use a standardized palpation procedure based on anatomical landmarks (count up from the sacral base and down from T12/ribs) and confirm with diagnostic ultrasound.	1 (1–4)	95.0^a^	0.0	5.0
	Ultrasound if available.	2 (1–3)	95.0^a^	5.0	0.0
	Palpation in a prone position in combination with ultrasound for verification.	2 (1–4)	85.0^a^	10.0	5.0
	Palpation of the spinous processes.	1.5 (1–4)	85.0^a^	10.0	5.0
	Place hands on iliac crests, identify the L4 spinous process, place a mark on the skin, go down towards the sacrum, identify the L5 spinous process, go up towards the thoracic vertebrae, identify each spinous process.	1 (1–4)	80.0^a^	10.0	10.0
	Having someone with sufficient experience landmarking spinous process perform the markings.	2.5 (1–5)	50.0	25.0	25.0
	Palpation, and confirmation by a healthcare professional.	3 (1–5)	40.0	20.0	40.0
	Check by palpation done by two people.	3 (1–5)	45.0	20.0	35.0
	Identify L5 via location 1st sacral tubercle (landing point). Then L5-S1 interspinous up to L1.	3 (1–5)	45.0	30.0	25.0
	L2 spinous process is at the level of the line joining the inferior borders of the 10th ribs. The intercostal line is at the level of the L3/4 interspinous space or L3 spinous process.	3 (1–4)	35.0	40.0	25.0
	It depends on the protocol, the type of study, and the research questions being asked if accurate palpation is needed.	3.5 (1–5)	35.0	15.0	50.0
Placing the wheels over the test area	Make sure that the wheels are aligned on the skin before running each trial.	1 (1–2)	100.0^a^	0.0	0.0
	Make sure there is enough vertical travel in the roller to test the most posterior part of the participants’ back.	1 (1–2)	100.0^a^	0.0	0.0
	Without changing the table height or moving the frame, move the roller wheels to the landing site by positioning the laser over the center of the “X” axis.	1 (1–2)	100.0^a^	0.0	0.0
	Jog wheel down onto participant and add enough cable slack (approximately 5 extra jogs down).	1 (1–2)	100.0^a^	0.0	0.0
	Move the roller wheels above the highest point of the test area.	1 (1–3)	95.0^a^	5.0	0.0
	Raise the plinth until the highest point on the participant is 3 cm from the wheels.	1 (1–3)	95.0^a^	5.0	0.0
	Some participants with hyper-lordosis may require more than 5 extra jogs down.	1 (1–4)	90.0^a^	5.0	5.0
Wheels starting position	Look at the laser from the same angle to ensure it is lined up perfectly before each trial.	1 (1–2)	100.0^a^	0.0	0.0
	Check the laser goes back to the reference point prior to subsequent runs.	1 (1–2)	100.0^a^	0.0	0.0
	Make sure the participant is not moving between the trials.	1 (1–2)	100.0^a^	0.0	0.0
	Mark the starting position with an “x”.	1 (1–4)	95.0^a^	0.0	5.0
	Photos of the back should be taken.	3 (1–5)	35.0	35.0	30.0
	Measure the length of the trajectory by a tape measure.	3 (1–5)	20.0	45.0	35.0
Instructions for participants during the assessment	You should relax your back and abdominals.	1 (1–2)	100.0^a^	0.0	0.0
	Let us know if you wish to stop the measurements at any time or if you have any concerns (e.g., discomfort).	1 (1–2)	100.0^a^	0.0	0.0
	You should remain still for the duration of the test (~ 15 min) even when you answer a question in between the trials.	1 (1–2)	100.0^a^	0.0	0.0
	You will be asked to hold your breath at various times during the procedure for approximately 10 s each time.	1 (1–2)	100.0^a^	0.0	0.0
	You should wait for my instructions before you move away from the table.	1 (1–2)	100.0^a^	0.0	0.0
	You should keep your arm position the same for the duration of the test.	1 (1–3)	95.0^a^	5.0	0.0
	You should not talk during the procedure.	1 (1–4)	90.0^a^	5.0	5.0
	The operator should check the participant’s readiness for each trial.	1 (1–4)	90.0^a^	5.0	5.0
	You’ll be instructed when you can start breathing again.	1 (1–4)	90.0^a^	5.0	5.0
	You should not endure discomfort at any time especially when adding weight plates during testing.	2 (1–4)	65.0	10.0	25.0
	You should give us a sign to indicate that you have exhaled the air and ready to be tested before each trial.	2.5 (1–5)	50.0	20.0	30.0
Instructions for participants after the assessment	You should contact us if you experience any discomfort in the next few hours or days.	1 (1–2)	100.0^a^	0.0	0.0
	Let us know if you feel discomfort after the session or any skin irritation. These two conditions might be expected, but they will eventually disappear.	1 (1–2)	100.0^a^	0.0	0.0
	Wait to get up until the device is removed from above you.	1 (1–2)	100.0^a^	0.0	0.0
	You may experience some mild, short-term pain and discomfort in the area that has been tested.	1 (1–4)	90.0^a^	5.0	5.0
	You may experience some dizziness. If so, sit for a few minutes before standing up.	1 (1–4)	90.0^a^	5.0	5.0
	It is normal to feel slightly stiff after the measurements.	1 (1–4)	85.0^a^	10.0	5.0
	Slowly get up and watch your head.	1 (1–4)	85.0^a^	5.0	10.0
	You might feel sore in the next 48 h, this is normal but if the pain does not subside after that time or you feel worried do not hesitate to contact the principal investigator.	2 (1–5)	75.0^a^	15.0	10.0
	No residue pain or discomfort should remain after the measurements. Any discomfort or problems should be reported to the staff at any time.	3 (1–5)	45.0	25.0	30.0
	You should walk on a level surface (low-level exercise) for a few minutes after the test procedure.	3.5 (1–5)	15.0	35.0	50.0
	No need for specific instructions after testing. Unless there is interest in the perception of stiffness or mobility in a given study.	4 (1–5)	10.0	15.0	75.0^a^
A good/bad trial definition	A good trial is a trial where the wheels follow the curvature of the spine without deviating sideways, and which does not cause discomfort to the participant.	1 (1–2)	100.0^a^	0.0	0.0
	A good trial is one in which the participant is relaxed, does not move, and holds his/her breath out for the entire trial.	1 (1–2)	100.0^a^	0.0	0.0
	A bad trial is the one with irregular change in the trajectory line.	1 (1–3)	90.0^a^	10.0	0.0
	A good trial is consistent data collected towards a single participant.	1.5 (1–4)	85.0^a^	10.0	5.0
	If the wheels did not move smoothly and they are not continuously pointed forward, it is a bad trial.	2 (1–4)	70.0^a^	20.0	10.0
	If the displacement decreased at a higher load, it’s a bad trial.	4 (2–5)	40.0	20.0	40.0
	In a good trial, the participant gets an appreciation of how the testing will feel.	4 (2–5)	10.0	30.0	60.0
	A good or bad trial would be defined based on patient reports and visual inspection.	4 (2–5)	10.0	25.0	65.0
	A good trial is when the same value is collected for all segments.	4 (2–5)	10.0	10.0	80.0^a^
	This is typically up to the participant whether the trial is good or bad.	5 (3–5)	0.0	10.0	90.0^a^
Instructions for the operator to ensure a good trial	I will monitor the wheels by enough cable slack and will align the wheels.	1 (1–2)	100.0^a^	0.0	0.0
	I will properly communicate with the participant what I expect from them and give them regular feedback.	1 (1–2)	100.0^a^	0.0	0.0
	I look for movement, breathing, and tonicity.	1 (1–2)	100.0^a^	0.0	0.0
	I will focus on the graphic trend.	1 (1–3)	95.0^a^	5.0	0.0
	I will double-check the data collected before letting the participants leave, repeat if failed.	1 (1–3)	95.0^a^	5.0	0.0
	I look at the graphics in the software after a few trials to make sure that the graphics look appropriate.	2 (1–5)	90.0^a^	5.0	5.0
	I will make sure that the graph output after each trial matches the general graph expected.	2 (1–5)	90.0^a^	5.0	5.0
	I’ll check the values.	2 (1–5)	85.0^a^	5.0	10.0
	If I noticed something different with the process, I would mark it as a bad trial.	2 (1–3)	80.0^a^	20.0	0.0
	It is necessary that the table on which the patient is positioned has armrests to rest the arms in prone position.	2 (1–5)	75.0^a^	15.0	10.0
Instructions for participants for between the measurement sessions	Use the restroom.	1 (1–2)	100.0^a^	0.0	0.0
	Maintain your normal routine.	1 (1–2)	100.0^a^	0.0	0.0
	Depending on what is being investigated, researchers might need to control for exercise, food intake, hydration levels (e.g., abdominal contents, gas, delayed onset muscle soreness, etc).	1 (1–3)	95.0^a^	5.0	0.0
	Activities between days depending on the research question.	1 (1–3)	90.0^a^	10.0	0.0
	Go for a walk.	1 (1–4)	80.0^a^	10.0	10.0
	You must not have any treatment on the spine between sessions unless this treatment is the subject of experimentation.	2 (1–4)	80.0^a^	15.0	5.0
	Recommendations to be more or less active than usual could be a confounding factor to results.	2 (1–4)	75.0^a^	15.0	10.0
	Do not begin new physically intensive activities between measurement sessions.	1 (1–3)	75.0^a^	25.0	0.0
	Do not do heavy weightlifting/training in between same-day sessions.	2 (1–4)	70.0^a^	25.0	5.0
	If you take medication like muscle relaxants or pain killers, take the medication after the assessment.	2 (1–5)	70.0^a^	15.0	15.0
	No strenuous exercise should be done in between sessions.	2 (1–4)	60.0	30.0	10.0
	Come back at the same time of the day.	2 (1–4)	60.0	15.0	25.0
	Don’t do any vigorous back exercises two days before the test.	2 (1–5)	55.0	25.0	20.0
	No additional care between sessions.	2.5 (1–4)	50.0	35.0	15.0
	Don’t undergo any physically demanding activity involving the back.	2.5 (1–5)	50.0	15.0	35.0
	Sleep well.	3 (1–4)	40.0	45.0	15.0
	Avoid big meals in between sessions.	3 (1–4)	35.0	50.0	15.0
	Avoid swimming and scrubbing your back.	3.5 (1–5)	15.0	35.0	50.0
	Wear the same clothes for the next session.	4 (2–5)	15.0	10.0	75.0^a^
Instructions for the same position over multiple measurement sessions	Use a permanent marker (particularly for S1) to ensure the starting position of the measurement is the same.	1 (1–2)	100.0^a^	0.0	0.0
	Keep the reference points intact.	1 (1–2)	100.0^a^	0.0	0.0
	Have a standardized examination table with markings that could be used to align participants in a reproducible manner.	1 (1–3)	95.0^a^	5.0	0.0
	Take a photo with the consent of the participant.	1 (1–4)	90.0^a^	5.0	5.0
	Put a band-aid/ adhesive tape on top of the marked “x” spot so you don’t lose it for the next visit.	1 (1–3)	85.0^a^	15.0	0.0
	Measure the trajectory distance.	2 (1–4)	70.0^a^	25.0	5.0
	Participants should feel just as comfortable as before.	2.5 (1–5)	50.0	20.0	30.0
	Since the testing plinth has a hole, the participant will always align at approximately the same distance from the cephalic end of the plinth.	3 (1–5)	45.0	40.0	15.0
	Take notes on the position of the patient (head, arms, legs).	3 (1–4)	40.0	40.0	20.0
	Tape on the table and the floor to ensure the same position of equipment and person on the table.	3 (1–5)	35.0	35.0	30.0
Software program crashes	I will stop the software and restart software.	1 (1–2)	100.0^a^	0.0	0.0
	I will inform the participant of the situation and will ask to lie still for the issue to be fixed.	1 (1–2)	100.0^a^	0.0	0.0
	I will ask the participant’s permission to start over.	1 (1–2)	100.0^a^	0.0	0.0
	I will ask participants if they would like a rest before starting over.	1 (1–2)	100.0^a^	0.0	0.0
	I will re-calibrate the device.	1 (1–3)	95.0^a^	5.0	0.0
	I will remove all the weights.	1 (1–3)	95.0^a^	5.0	0.0
	Make sure the participant is safely out of the device.	1 (1–3)	95.0^a^	5.0	0.0
	I will remove the device from the above participant and start over.	1 (1–5)	85.0^a^	10.0	5.0
	My actions depend on the severity of the crash. For example, if I have to recalibrate the trajectory, I will have to recollect all trials.	1.5 (1–4)	75.0^a^	15.0	10.0
	I will re-do the problematic trial and resume the measurements.	2 (1–4)	70.0^a^	15.0	15.0
	I will re-do the measurements from 0 N.	2 (1–4)	65.0	25.0	10.0
	Software program crashes are less likely to be related to the control box issue. Therefore, turning off the computer or control box will be my last resort.	2 (1–4)	65.0	25.0	10.0
	I will close the software and restart the computer.	2 (1–5)	55.0	30.0	15.0
	I will turn off the control box and restart the whole system.	2 (1–5)	55.0	15.0	30.0
	I will re-schedule the participant.	4 (1–5)	15.0	15.0	70.0^a^
	I will press the emergency stop button.	4 (1–5)	10.0	30.0	60.0
Participants’ Safety	The safety stop button should immediately elevate the load and return the rolling arm to a position away from the patient - so that the patient can exit if needed.	1 (1–2)	100.0^a^	0.0	0.0
	Clear instructions to participants with expectations explained.	1 (1–2)	100.0^a^	0.0	0.0
	The participants should not get up before the frame is off them.	1 (1–1)	100.0^a^	0.0	0.0
	Make sure the device is properly operational (or locked in place) when loading weights.	1 (1–2)	100.0^a^	0.0	0.0
	Make sure all the 1 kg weights are removed from the device before and after assessment by the VerteTrack.	1 (1–1)	100.0^a^	0.0	0.0
	Familiarize yourself with the location of the hardware emergency stop (E-stop) before assessment by the VerteTrack.	1 (1–2)	100.0^a^	0.0	0.0
	Follow the suggested pre-test protocol to make sure all “detectors” are functioning properly.	1 (1–1)	100.0^a^	0.0	0.0
	Procedures explained to participants for emergency stop.	1 (1–2)	100.0^a^	0.0	0.0
	Continuing to check in with the patient throughout the process to make sure that they are feeling okay.	1 (1–2)	100.0^a^	0.0	0.0
	Disinfect the wheels/bench/equipment prior to each participant.	1 (1–1)	100.0^a^	0.0	0.0
	Make sure to remove the weights one by one at the end of the measurement.	1 (1–4)	95.0^a^	0.0	5.0
	Have an easy reading format for clients with disabilities before assessment by the VerteTrack.	1 (1–4)	90.0^a^	5.0	5.0
	Make sure to depress the emergency stop and then disengage it to ensure it is working before assessment by the VerteTrack.	1 (1–4)	85.0^a^	5.0	10.0
	I will raise the plinth when not testing to make sure it will not drop if it malfunctions.	3 (1–5)	45.0	35.0	20.0
	Have a mirror to be able to see the client’s face.	3 (1–5)	10.0	50.0	40.0

Note: Scores are on a scale from 1 to 5, where 1 = strongly agree, 2 = agree, 3 = neither agree or disagree, 4 = disagree, and 5 = strongly disagree

Consensus was achieved when at least 70% of participants strongly agreed/agreed or strongly disagreed/ disagreed with a statement

^a^Consensus reached

**Table 3:** The number of consensus statements under each category

Category	Number of consensus statements	Number of non-consensus statements	Total number of statements
Inclusion criteria	2 (50.0%)	2 (50.0%)	4
Exclusion criteria	11 (47.8%)	12 (52.2%)	23
Pregnancy time frame limitation	0 (0.0%)	7 (100%)	7
Familiarization procedure	8 (66.7%)	4 (33.3%)	12
Instructions for participants before the assessment	10 (90.0%)	1 (9.1%)	11
Identification of spinous processes	5 (45.5%)	6 (54.5%)	11
Placing the wheels over the test area	7 (100%)	0 (0.0%)	7
Participants’ starting position	4 (66.7%)	2 (33.3%)	6
Instructions for participants during the assessment	9 (81.8%)	2 (18.2%)	11
Post-test instructions	9 (81.8%)	2 (18.2%)	11
Definitions for a good or bad trial	7 (70%)	3 (30.0%)	10
Procedures to ensure a good trial	10 (100%)	0 (0.0%)	10
Instructions for between-session assessments	11 (57.9%)	8 (42.1%)	19
Instructions for reaching the same position in case of multiple assessments	6 (60%)	4 (40.0%)	10
Software program crashes	11 (68.8%)	5 (31.3%)	16
Optimizing participant safety	13 (86.7%)	2 (13.3%)	15
Total	123	60	183

## Discussion

In this Delphi study, 20 panelists reached consensus on the majority of items relating to VerteTrack spinal stiffness measurements covering a wide range of domains including recruitment criteria, familiarization procedure, instructions for participants/ operators, technical issues, and safety. This is the first time, to our knowledge, that consensus has been used to obtain a common protocol on instrumented spinal stiffness measurements.

It is important to stress that the key feature of the approach used in this study is the consensus of individuals in the field of spinal manipulative therapy and low back pain research who had experienced working with VerteTrack. Therefore, the intent was not to find “the best” protocol for measuring spinal stiffness or to present an instrument as “the only” mechanical method for measuring spinal stiffness. Our goal was to develop a standard protocol for measuring spinal stiffness using a loaded rolling wheel device that could be used as a common resource in future studies.

The surveys identified some previously known considerations when measuring stiffness including the participant’s testing position, trunk muscles contraction, intra-abdominal pressure, respiratory cycle, and relocation of target spinal landmarks [4, 5]. This supports the quality and validity of our participants’ answers as these items have been developed over years in this field and the literature. For instance, one of our participant’s recommendations was to ask the patient to relax their back muscles during the assessment which is in line with an early study that showed spinal extensor muscle activities could induce changes in the mechanical responses to posteroanterior stiffness testing [20]. Furthermore, the surveys identified other factors not described previously in the literature including optimizing participant’s safety, a definition for a good/ bad trial, procedures to ensure a good trial, placing the device over the test area, instructions for reaching the same position in case of multiple assessments, and fixing software program crashes. This emphasizes the importance of group opinion over that of individuals for bringing new topics into focus that can be validated and studied in future works.

Interestingly, there was one specific area where no agreement was reached: the exclusion of pregnant participants from spinal stiffness measurements. One explanation for this lack of agreement is that different respondents may have different experiences in this area through diverse research designs that would, or would not, allow participants to be enrolled at different stages of pregnancy. This speculation is supported by studies to date that have employed VerteTrack. Of six studies using VerteTrack in human participants to date, three excluded pregnant participants [11, 21, 22], one excluded pregnant participants in the second or third trimester of pregnancy [10] and the remaining studies did not mention pregnancy at all [23, 24].

All items for which consensus was reached were consolidated into a final best practice protocol (Additional file 1) for using the VerteTrack. The resulting standard protocol is expected to improve the accuracy and efficiency of spinal stiffness measurements using the VerteTrack, facilitate the training of new operators, increase consistency of these measurements in multicenter studies, and finally provide the synergy and potential for data comparison between spine studies internationally. Our final protocol provides directions for researchers and clinicians who use the VerteTrack to measure spinal stiffness. However, caution should be used if between-patient comparisons are made (for many reasons including differences in plinth rigidity as well as between-person variations). The final protocol could be useful for other technologies that assess stiffness and even manual assessment of spinal stiffness. We encourage researchers in this area to review this protocol and consider adopting it for their own purpose. While the technical part of the protocol explaining how to operate the device may not be useful for manual assessments or devices that test participants in sitting position, however, some general information for spinal stiffness measurements has been provided and may be of benefit.

### Strengths and limitations

The strengths of this study include the development of a consensus-based protocol based on 80% of the global population of persons with VerteTrack training and experience for Round 1 and 100% follow-up responses for Rounds 2 and 3. The relative heterogeneity in our participants may enhance the generalizability of the protocol and may have ensured that a greater spectrum of opinions was considered. The initial pilot survey improved the structure and readability of the questions before executing the full-scale project. In addition, Round 1 of our Delphi study provided the possibility of open responses and gave the participants the freedom to elaborate on the research topic which may increase the richness of the data collected. Although author bias cannot be completely eliminated from this type of research, it was minimized through implementing a Delphi consensus process using anonymous participant ratings and comments. The deidentification anonymity of participants’ answers to the questions also provided more open and honest feedback and prevented response bias.

It is acknowledged that the Delphi method itself has inherent limitations including Level V in the hierarchy of evidence-based medicine and the small sample size required. Although the final protocol was developed based on Delphi participants’ responses to 3 rounds of questions, it was not distributed to them for approval at the end of the study. Further, lack of interaction between participants in the Delphi (e.g., face-to-face meetings) may deprive panelists of exchanging important information, such as clarification of reasons for disagreements.

## Conclusions

Using a Delphi approach, a consensus-based protocol for measuring spinal stiffness using the VerteTrack was developed. This standard protocol was designed to i) improve the accuracy, efficiency, and safety of spinal stiffness measurements using the VerteTrack, ii) facilitate the training of new operators iii) increase consistency of these measurements in multicenter studies, and iv) provide the synergy and potential for data comparison between spine studies internationally.

## Supplementary Information

**Additional file 1.** VerteTrack Operations Manual (Chapter 1: General information. Chapter 2: Device Operation. Chapter 3: Practical Considerations).

## Data Availability

The datasets used and analyzed during the current study are available from the corresponding author on reasonable request.

## Abbreviations

LBP: Low Back Pain
REDCap: Research Electronic Data Capture
