# Supplementary material for: A consensus approach toward the standardization of spinal stiffness measurement using a loaded rolling wheel device: results of a Delphi study
Source: BMC Musculoskelet Disord. 2021 May 13;22:436. doi: 10.1186/s12891-021-04313-6 (PMC8120899; doi:10.1186/s12891-021-04313-6)
Supplement: Supplementary file 1 — Additional file 1. VerteTrack Operations Manual (Chapter 1: General information. Chapter 2: Device Operation. Chapter 3: Practical Considerations). [file 12891_2021_4313_MOESM1_ESM.pdf]

# VerteTrack

## Operations Manual

April 24, 2021

# Table of Contents


*CHAPTER 1: GENERAL INFORMATION* .......... *4*

1-1 Test population .......... 4

1-2 Physical Description .......... 4

1-3 Operational Summary .......... 5

1-4 Clinical Applications .......... 5

1-5 Inclusion Criteria .......... 5

1-6 Exclusion Criteria .......... 6

*CHAPTER 2: Device Operation* .......... *7*

2-1 Pre-testing Checklist .......... 7

2-2 Device Preparation .......... 7

2-3 Check Limit Switches and Jogs .......... 8

2-4 Home the Device .......... 10

2-5 Participant Welcoming Activities (Participant Testing, Page 1) .......... 11

2-6 Determining Wheel Size and Inter-Wheel Distance .......... 11

2-7 Mark the Participant (Participant Testing, Page 1) .......... 12

2-8 Positioning the Wheels over the Test Area (Participant Testing, Page 1) .......... 13

2-9 Teach FWD Trajectories (Participant Testing, Page 1) .......... 14

2-10 Select the Wheel Spacing and Wheel Size .......... 15

2-11 Name FWD and REV Trajectory Files .......... 15

2-12 Load the Filenames (Participant Testing, Page 2) .......... 15

2-13 Run the FWD Trial (Participant Testing, Page 2) .......... 16

2-14 Return the Wheels by Running the REV Trial (Participant Testing, Page 2) .......... 18

2-15 Check the Trial, Save the Trial (Participant Testing, Page 2) .......... 18

2-16 A Good/Bad Trial Definitions .......... 19

2-17 Instructions for Operator to Ensure a Good Trial .......... 19

2-18 Collecting the Next Trial .......... 20

2-19 End Data Collection with this Participant .......... 20

2-20 Testing Another Participant .......... 20

2-21 If You Are Finished with this Session .......... 21

2-22 If You Stopped the Software and Want to Start Again .......... 21

2-23 Troubleshooting ... 22

*CHAPTER 3: Practical Considerations ... 24*

3-1 Participant Briefing (pre-test) ... 24

3-2 Example Remarks to Participant (pre-test) ... 24

3-3 Participant Briefing (during the test) ... 25

3-4 Example Remarks to Participant (during the test) ... 26

3-5 Participant Briefing (post-test) ... 26

3-6 Example Remarks to Participant (post-test) ... 26

3-7 Participant Briefing (between tests) ... 27

3-8 Example Remarks to Participant (between tests) ... 27

3-9 Recommendations for Operators to Reach the Same Position over Multiple Measurement Sessions ... 27

3-10 Summary of Recommendations for Operators to Optimize Participant Safety ... 28

# CHAPTER 1: GENERAL INFORMATION

This chapter describes general information about the device including its intended test population, operational requirements, and different components.

### 1-1 Test population

The VerteTrack device is a biomedical device designed to measure posteroanterior mobility of the thoracic and lumbar spine. This device may only be operated by trained individuals who are familiar with human spine anatomy and within a research study having ethical approval.

### 1-2 Physical Description

The VerteTrack device is a software-driven mechanical device that incrementally applies force to acquire, process, and display stiffness data.

The device is composed of 6 major components:

1. A cube-shaped aluminum frame (Width 1080 mm × Height 1090 mm × Length 1510 mm) with lockable casters. **Figure 1-1 (A)**
2. A custom-made wheel apparatus consisting of a vertical rod, two rolling wheels, and a laser pointer. **Figure 1-1 (B)**
3. A stepping motor system along X, Y, and Z axes with built-in encoders (resolution = 0.007 mm) (National Instruments, USA). **Figure 1-1 (A)**
4. A string potentiometer (resolution = 0.020 mm, TE Connectivity, USA).
5. Custom-made LabVIEW software used to operate the device (National Instruments, USA). **Figure 1-1 (A)**
6. Two emergency stop buttons (software and hardware) allowing the operator to stop the measurements at any time.

*Note:* **Always** use the software emergency stop first if needed before using the hardware stop. This is because the software stop will withdraw the wheels from the participant which is the desired way to abort testing. If the hardware stop is used, power to the motors is killed which will result in the current weight of the wheels being suddenly dropped, possibly on to the participant.

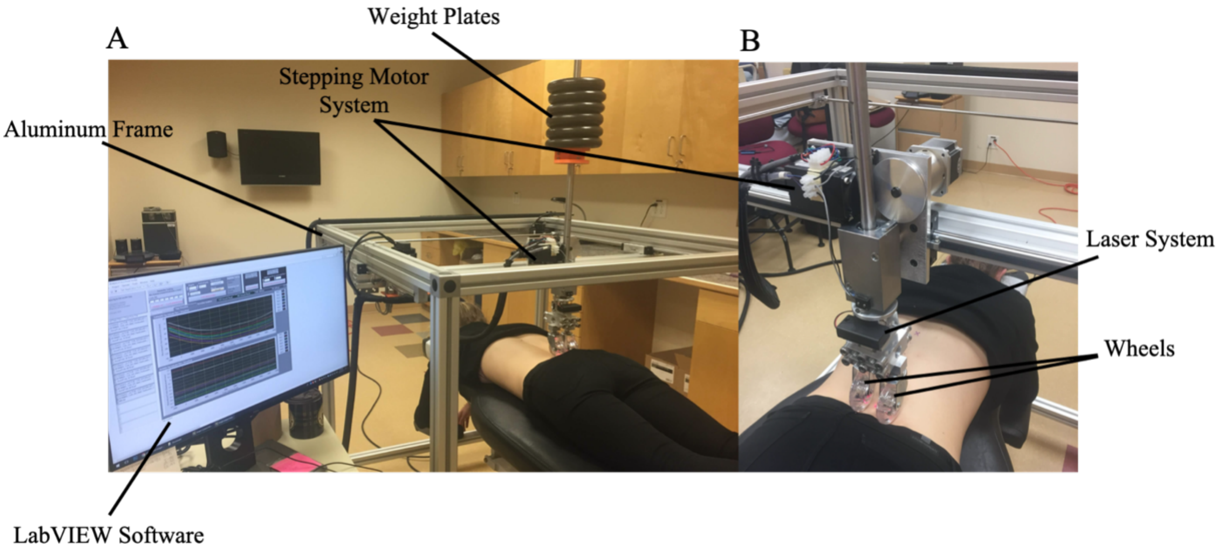


**Figure 1-1.** The VerteTrack Device: (A) The device components; (B) Roller Apparatus

## 1-3 Operational Summary

In brief, the movement of the load-bearing wheels is mapped out as a horizontal trajectory (XY) by manually moving the wheels to trace a path on the participant's spine with the laser system. Following that trajectory, a pre-selected vertical load is continuously applied by the wheel apparatus while the resulting tissue deformation is measured by a wire string potentiometer. The string potentiometer measures the vertical position of the wheel apparatus and provides real-time feedback to the control system. The additional load can then be added to the wheels in increments of 10 N (Maximum = 60 N) using weight plates. Signals from the encoders that record the positions of the motors are collected by customized LabVIEW software at a collection rate of 200 Hz. The resulting force-deformation profile of the targeted segments (e.g., lumbar segments) can then be used to generate estimates of bulk stiffness along the trajectory.

## 1-4 Clinical Applications

- Thoracic spine
- Lumbar spine

## 1-5 Inclusion Criteria

- The participant's ability to tolerate a load of at least 40 N while in prone lying.

- Body mass index (BMI) under 40 for ease of locating spinous processes which define the wheel trajectory.

### 1-6 Exclusion Criteria

- Pregnancy.
- Skin lesions, infections, or open wounds in the vicinity of where the wheels will follow their trajectory (i.e., the skin surface of the lumbar and/or thoracic spine).
- An inability to lie in the prone position (e.g., severe deformities to spine or limbs, static tremor, uncontrolled epilepsy, etc.).
- Serious spinal pathology (e.g., spinal tumor, fracture, infectious disorder, osteoporosis, or other bone demineralizing condition, etc.).
- An inability to sustain held, active expiration (functional residual capacity) for at least 10 seconds.
- An inability to follow instructions (e.g., those with dementia or participants who may move during the test (e.g., age under 18)).
- A head, neck, or thoracoabdominal surgery within the last 6 months.
- Unstable spondylolisthesis.
- Unstable and/or acute disc herniation or injury.
- People who do not feel comfortable with the VerteTrack procedure.
- Unstable heart conditions.

# CHAPTER 2: Device Operation

This chapter will describe practical considerations for VerteTrack users. Operators will gain a fundamental appreciation of participants' needs, recording technique, device operation, feedback training, and data management. The purpose of this chapter is to ensure the correct and safe operation of the VerteTrack device (hardware, software, and mechanical).

## 2-1 Pre-testing Checklist

Before operating the device, make sure to:

- Remove each weight from the device.
- Familiarize yourself with the location of the hardware emergency stop (E-stop). Before using the device, test the emergency stop by depressing and then disengaging it. Ensure the E-stop is disengaged before use – this is a common reason why motors may not operate when the machine is first operated – someone has depressed the E-stop.
- Never step on or run over the device's cables which are custom-made and fragile.
- Wipe the wheels with a proper disinfectant (e.g., soapy water, alcohol).
- Ensure there are no cracks or other damage to the wheels before they are used. Inspect the wheels' surfaces for cracks and feel for cracks with fingertips as well.

*Caution:* **Do not** use wheels if they are cracked, damaged, or broken.

## 2-2 Device Preparation

- Lower the plinth completely using the plinth control. **Figure 2-1**
- Roll the frame away from the plinth. **Figure 2-2**
- Turn on the device computer and log in.
- Turn on the device control box. **Figure 2-3**
- Launch the device software located on the desktop (Swiper Control V07.1 Compiled APPLICATION.EXE.). **Figure 2-4**

*Note:* The device control box must always be turned on before launching the software. If the computer software was running before the control box was turned on, close the software, turn off the control box, then turn on the control box, and restart the software.

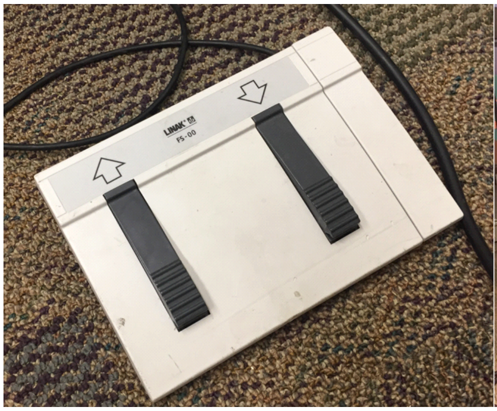

**Figure 2-1.** Plinth Control (example)

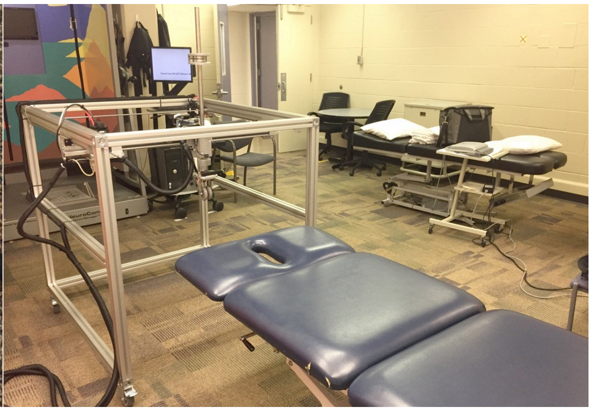

**Figure 2-2.** Frame away from the plinth.

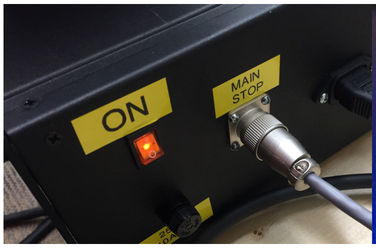


**Figure 2-3.** Turn the control box on

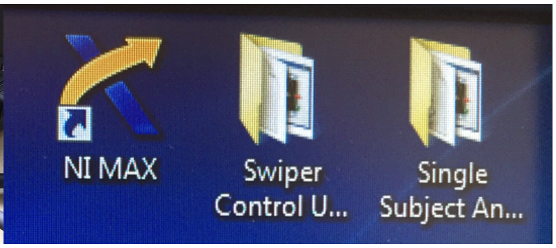


**Figure 2-4.** Launch Program

## 2-3 Check Limit Switches and Jogs

- Click the Check Limit Switches button in the software. **Figure 2-5**
- Depress each of the 6 limit switches and ensure the software light turns on for each sensor. Make sure the correct light turns on for the correct sensor.
- Click the Stop Checking button to end checking the limit switches. **Figure 2-6**
- At the top of the software, use the jog buttons to move the device in all 6 directions. **Figure 2-7**

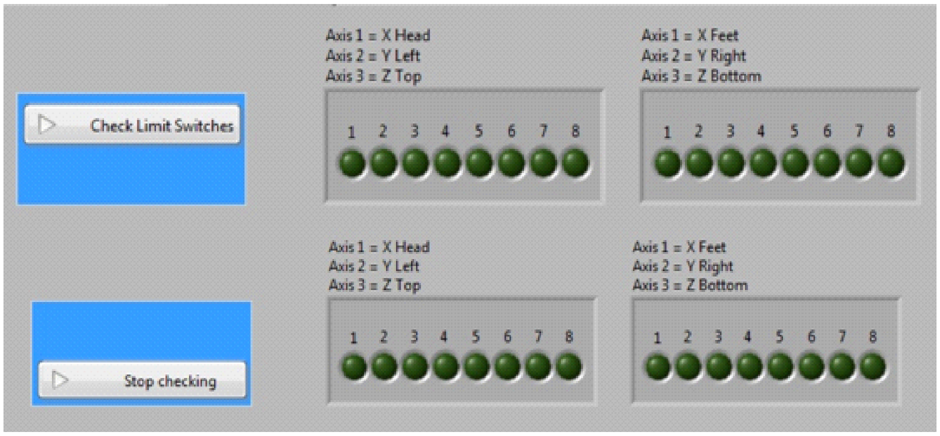


Figure 2-5. Limit switches in the software

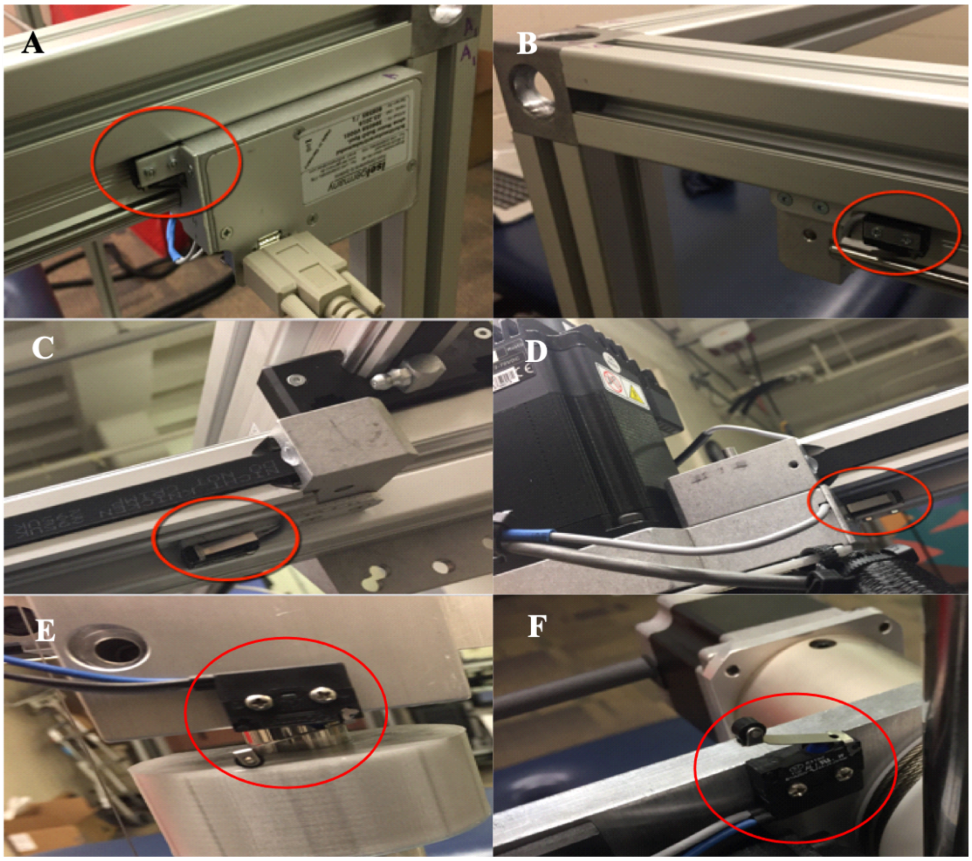


Figure 2-6. Mechanical limit switches: A) X-axis head, B) X-axis feet, C) Y-axis left, D. Y-axis right, E) Z-axis bottom, F) Z-axis top

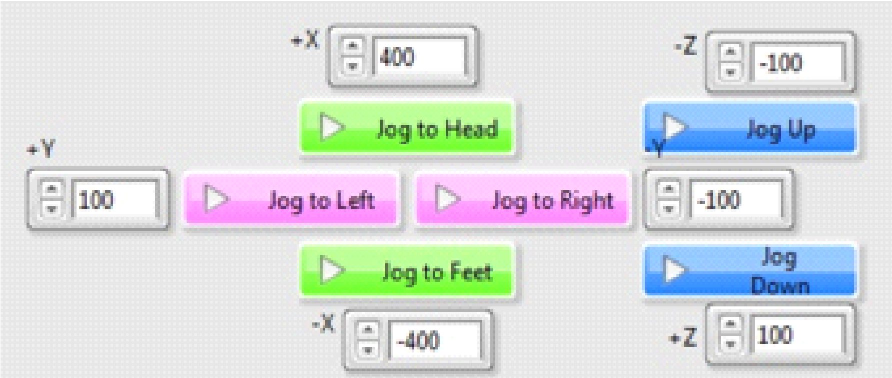


**Figure 2-7.** Jog buttons at the top of the software

## 2-4 Home the Device

- Before homing each axis, make sure that each direction of travel is clear of cables or other obstacles. Only run one homing direction at a time.
- Home the X-axis. **Figure 2-8**
  - Observe the process and watch for any cable impingement.
  - Wait for homing to complete – do not start any other processes
- Home the Y-axis. **Figure 2-8**
  - Observe the process and watch for any cable impingement.
  - Wait for homing to complete – do not start any other processes
- Home the Z-axis. **Figure 2-8**
  - Observe the process and watch for any cable impingement.
  - Wait for homing to complete – do not start any other processes

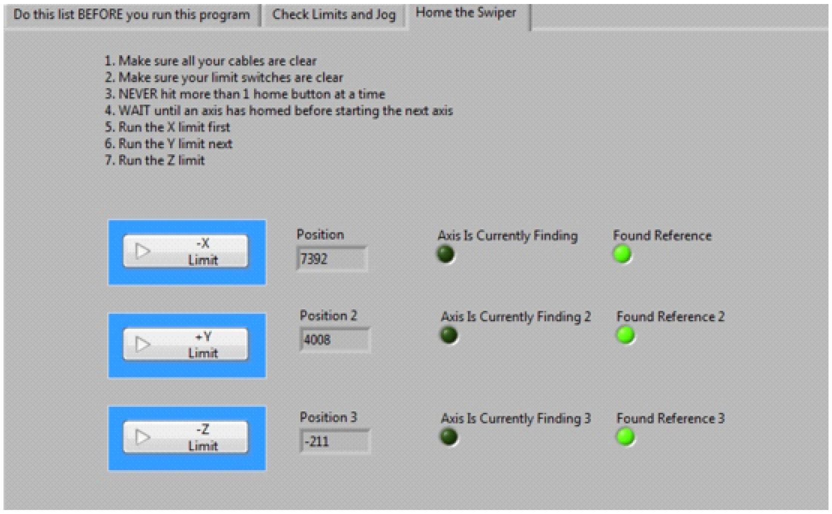


**Figure 2-8. Home the device**

## 2-5 **Participant Welcoming Activities** (Participant Testing, Page 1)

- Complete any forms for the study as much in advance as possible.
- Before testing begins, ensure the participant has gone to the restroom.
- Familiarize the participant with the procedure (See Chapter 3).

## 2-6 Determining Wheel Size and Inter-Wheel Distance

- For most participants, use the largest diameter wheels (3.0 inches).
- Before the participant is under the machine, examine their back and measure the width of the paraspinal tissues. **Figure 2-9**

*Note:* The wheels **should not** run on the spinous, nor too close to the spinous to avoid pulling the skin down over the spinous itself. Ideally, the wheels run over the top of the paraspinal tissues and above the lamina/facets. The wheels should be set no wider than the paravertebral tissues.

- Select the inter-wheel distance that best matches the distance below, or the next biggest size. For most adults, 1.125 inches (29mm) will suffice.
- If necessary, remove wheels with supplied wrench and change position.
- **If changing the wheels, or the inter-wheel distance, be careful not to twist wheel housing. This will potentially break the string potentiometer.**

Table 2-1. Wheel position with the corresponding distance between inner wheel face

| **Wheel position** | **Distance between inner wheel face** | |
|---|---|---|
| Position 1, closest 2 holes | 0.625 inches | 16 mm |
| Position 2 | 1.125 inches | 29 mm |
| Position 3 | 1.625 inches | 41 mm |
| Position 4, furthest 2 holes | 2.125 inches | 54 mm |

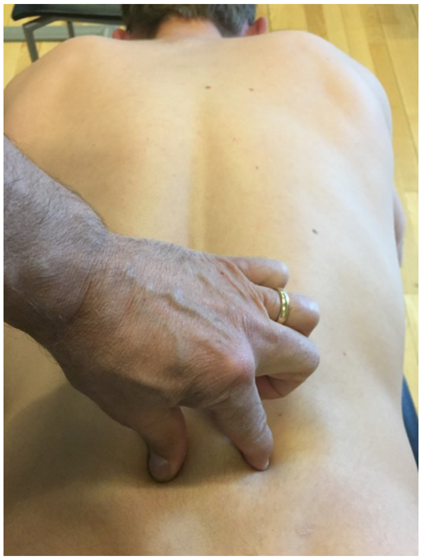

**Figure 2-9.** Gauging the width of spinous tissues

**2-7 Mark the Participant** (Participant Testing, Page 1)

- Place the participant in prone position on the plinth. Add foam roller under their legs.
- If using palpation, use a standardized palpation procedure based on anatomical landmarks (e.g., count up from the sacral base and down from T12/ribs).
    - Place hands on iliac crests, identify the L4 spinous process, place a mark on the skin, go down towards the sacrum, identify the L5 spinous process, go up towards the thoracic vertebrae, identify each spinous process.
    - Mark the starting position with an "x". **Figure 2-10**
    - Confirm with diagnostic ultrasound if available.
- If possible, use ultrasound to begin with for better accuracy in identifying spinous processes.

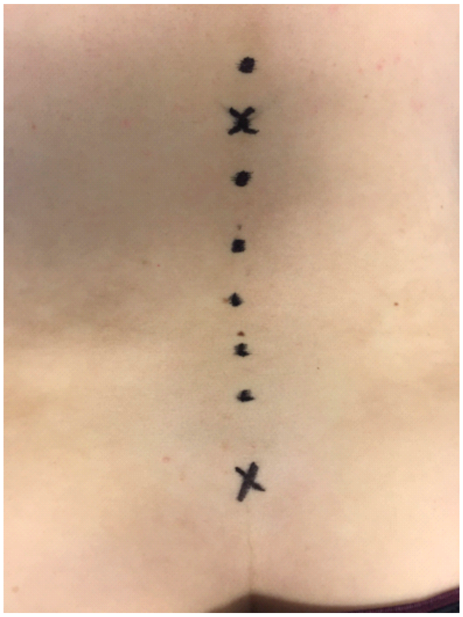

**Figure 2-10.** Mark the participant

**2-8 Positioning the Wheels over the Test Area** (Participant Testing, Page 1)

- Clean the wheels for this participant.
- Make sure the plinth is at the lowest setting
- Roll the frame back over the participant so the middle of the frame is aligned T7.
- **Lock the wheels of the frame.**
- Tell the participant to **remain still** for the rest of the testing session.
- **Make sure to keep the arm position of participants the same – whatever you decide.** If possible, it is preferred to have the arms resting on supports that are part of the plinth itself.
- In software, click the Kill XY Axes Button.
    - If one of the axes becomes locked and cannot move, you likely ran into the limit switch. Kill axes again and move the wheels manually to the middle of the frame. **Figure 2-11**
- With the axes being killed, move the device manually so that the wheels are above the **highest point of the test** area (most posterior).
    - Lumbar spine – Likely the base of sacrum but check T/L region.
    - Thoracic spine – Apex of kyphosis.

- **Ensure there is enough vertical travel space for the wheels to test the most posterior part of the participant's back.**

- **Only now, raise the plinth** until the highest point on the participant is 3 cm from the wheels. **Figure 2-1**

***Note:* From this point on, do not change the plinth height for this participant.**

- Without changing the plinth height or moving the frame, move the wheels to the landing site by positioning the laser over the center of the "X".
- Make sure that the wheels are aligned in the direction of travel on the skin before running each trial.

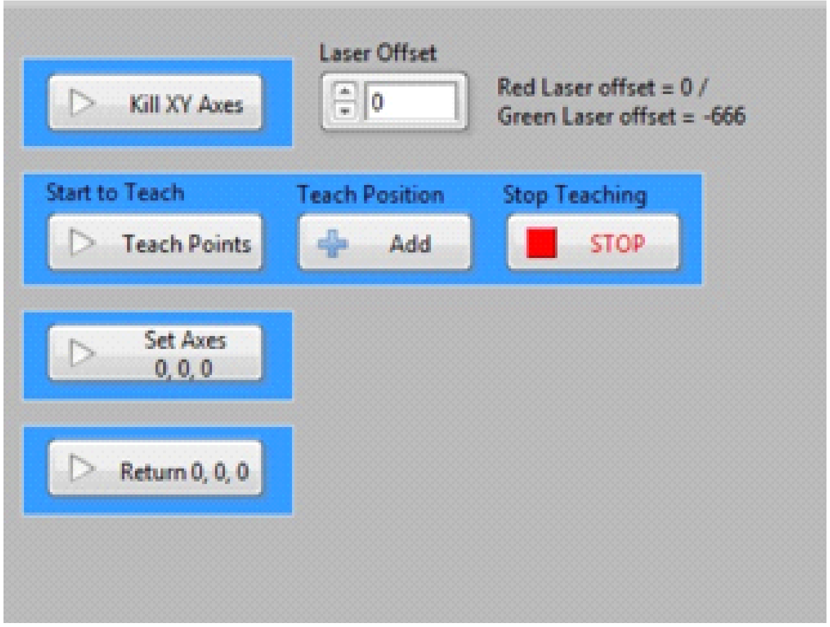


**Figure 2-11.** Kill Axes, Teach Points, Set Axes 000, Return 000

## 2-9 Teach FWD Trajectories (Participant Testing, Page 1)

- Turn on the laser.
- Without changing the plinth height or moving the frame, move the wheels to the Landing Site by positioning the laser over the center of the "X".

*Note:* When placing the laser, make sure to do so when the participant breathes out. The location of the skin markings can change quite a bit at different parts of the respiratory cycle.

- Click the Teach Points button. **Figure 2-11**
- Click the Add Point button immediately to capture 0, 0, 0 at the Landing Site. **Figure 2-11**

- Now move the laser manually to the next point and click the ADD A POINT button.
- Repeat this until all the points have been added including the “X” of the Lifting Point.
- When you are done, press the Stop Teaching button.

### 2-10 Select the Wheel Spacing and Wheel Size

- From the chart, pick the correct entry that describes the wheel spacing and wheel size. If using the largest wheel and second position, select the second choice (3.0 ICE, 1.125).
- Once you select the correction wheel diameter/position setting, press SET FILENAME.

### 2-11 Name FWD and REV Trajectory Files

- You will now be asked to name the FWD trajectory file. Use the participant's name or ID (whichever is appropriate) and include the letters FWD in the trajectory name. Save to the Desktop.
- You will now be asked to name the REV trajectory file.
    - Always save to the Desktop.
    - Use the participant’s name or participant ID (whichever is appropriate) and include the letters FWD in the trajectory name.
    - To avoid any startle, **let the participant know that as soon as you enter the rev filename, the device will move to the 0, 0, 0 position.**
- Click the Set Axes to 0,0,0 button. **Figure 2-11**
- Click the Return to 0,0,0 button. **Figure 2-11**
- Turn off the laser.

### 2-12 Load the Filenames (Participant Testing, Page 2)

- In the Data Collection Tab of the software, Load the filename of the FWD file in the appropriate text box. Do this by clicking on the file folder icon beside the text box. **Figure 2-12**
- Load the filename of the REV file in the adjacent text box. Do this by clicking on the file folder icon next to the text box.

- In the Participant File Name box, use the file folder icon to load a pre-existing trajectory file from the desktop. This will ensure that the correct path is loaded to the desktop. Then, change the filename in the textbox to follow the study's file naming protocol.

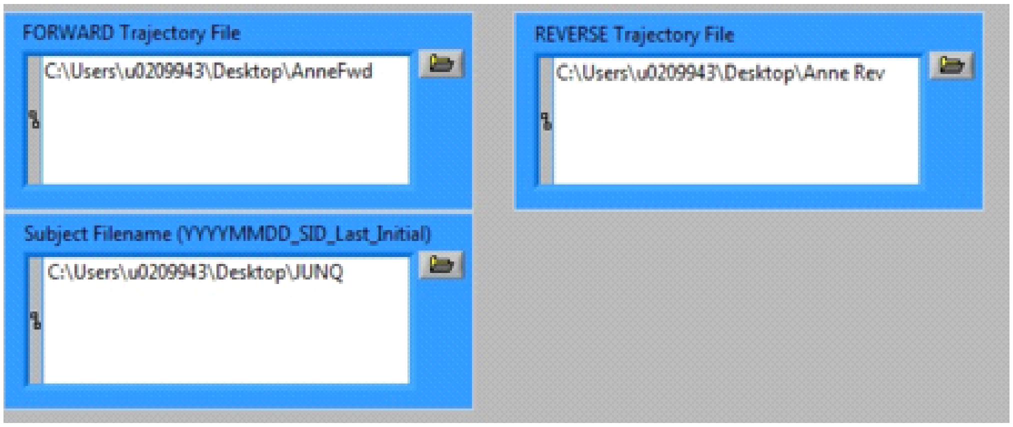


**Figure 2-12.** Load Filenames

**2-13 Run the FWD Trial** (Participant Testing, Page 2)

- Familiarize yourself with the Software Stop button and the Hardware E-stop. **Figure 2-13**
- Re-emphasize that the participant is to remain still during data collection.
- Add a 1 kg weight if needed (always add the additional 1 Kg weights to the device one at a time).
- Click the Start Trial button. **Figure 2-14**
- Review breathing with the participant if needed.
- Tell the participant that you are moving the wheels of the device down to touch their back.
- Using the newly appearing jog buttons (not the ones at the top of the screen), jog wheels down onto the participant and add enough cable slack (approx. 5 extra jogs down).
- Some participants with hyper-lordosis may require more than 5 extra jogs down.
- Align the wheels in the direction of travel before each trial if needed. **Figure 2-15**

- Look at the laser from the same angle to ensure it is lined up perfectly before each trial.
- When the wheels are fully touching the participant's skin and they are settled under the applied mass, check if the participant is ready, then start the breathing protocol and run the trajectory. **Figure 2-14**
- When the test is completed, the wheels will lift on their own.
- Now you can instruct the participant to breathe again.

*Note*:

- Do not leave the weight on the participant any longer than necessary. As soon as it settles on their skin and enough slack is provided, begin breathing and run the trajectory.
- Always consider the first round as a practice round to let the participant feel the testing procedure.

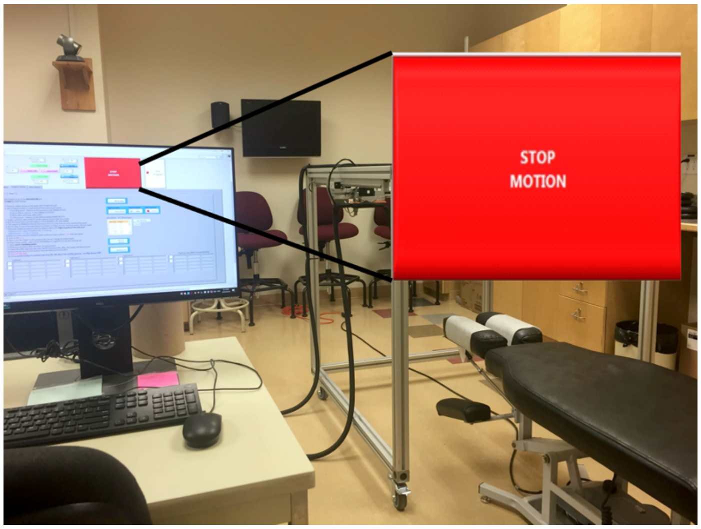


**Figure 2-13**. Software Emergency Button

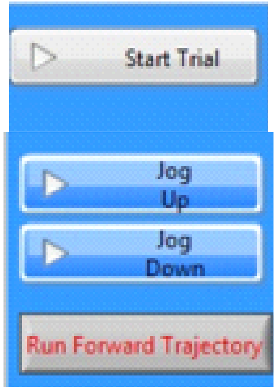


**Figure 2-14.** Start trial, Trial Jog, Run Trajectory

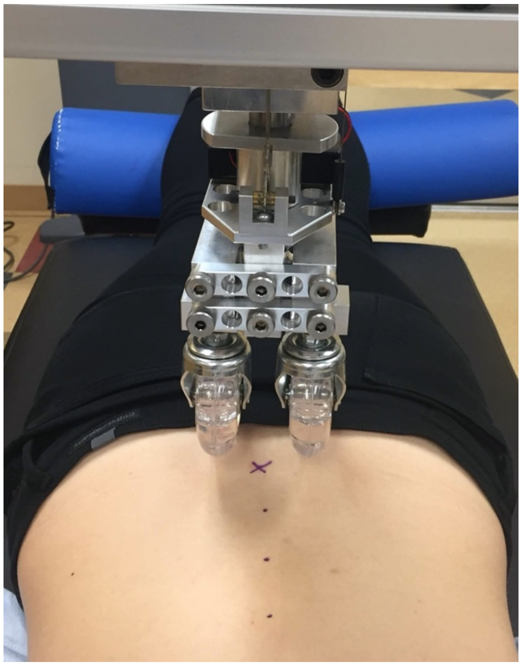

**Figure 2-15.** Make sure the wheels are aligned in the direction of travel before each trial

**2-14 Return the Wheels by Running the REV Trial** (Participant Testing, Page 2)

- Run the trajectory and the reverse trajectory will complete without the wheels touching the participant.
- Make sure the laser goes back to the reference point prior to subsequent runs.

**2-15 Check the Trial, Save the Trial** (Participant Testing, Page 2)

- Watch the participant during the trial to see if they breathe, move, perform a Val Salva or voluntarily contract their muscles. This may be a sign of discomfort.
- Check the data graph of the trial to ensure there were no breathing or movement artifacts in the collected data.
- Update the filename as appropriate. **Figure 2-16**

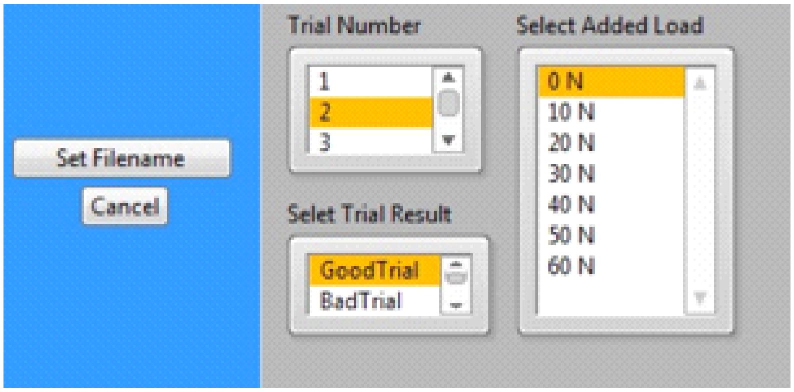


**Figure 2-16.** Revise trial filename

## 2-16 A Good/Bad Trial Definitions

- A good trial is a trial where the wheels follow the curvature of the spine without deviating sideways, and which does not cause discomfort to the participant.
- A good trial is one in which the participant is relaxed, does not move, and holds their breath for the entire trial.
- A good trial is one with consistent data collected for a single participant
- A bad trial is one with irregular change in the trajectory line.
- A bad trial is one where the participant breathes, moves, contracts muscles or contains any other event that would not occur in a normal test.
- If the wheels did not move smoothly and were not continuously pointed forward, it is a bad trial.

## 2-17 Instructions for Operator to Ensure a Good Trial

- Make sure you provide enough cable slack before the wheels move on the back.
- Make sure to align the wheels in the direction of travel.
- Communicate with the participant what you expect from them and give them regular feedback.
- Look for participant movement, breathing, and tonicity.
- Don't forget to check the graphical readout of the data after each trial.

- Double-check the data collected and make sure you have all your files before letting the participants leave. Repeat testing as needed.
- Look at the data graphs after each trial to make sure the data looks appropriate.
- Check the displacement values.
- If you noticed something different with the process, mark it as a bad trial.
- It is highly recommended that the plinth on which the participant is positioned has armrests to rest the arms in prone position.

## 2-18 Collecting the Next Trial (Participant Testing, Page 2)

- Make sure to align the wheels and the wheel housing before the next trial.
- Make sure the participant does not move between the trials (to talk to you, scratch, etc.).
- Go to Run the START TRIAL step.

## 2-19 End Data Collection with this Participant

- Tell the participant that the testing has ended but caution the participant not to get up until you tell them.
- Lower the participant plinth.
- Unlock frame wheels and roll frame away from the plinth.
- Help the participant to sit on the plinth remembering they may have difficulty after being prone. Ensure they do not bump their head on anything when arising from the plinth.
- Make sure the participant sits first on the plinth and is not dizzy before they stand up.
- Stand by to assist the participant as they stand.
- Thank the participant and give them any instructions for further testing etc.
- Now that the participant has left, remove weights from the device **one at a time**.

## 2-20 Testing Another Participant (After Participant)

- Go back to "Welcome Activities", Tab 1, Page 1

### 2-21 If You Are Finished with this Session (After Participant)

- Stop the program using the Big white software button. **Figure 2-17**
- **Always make sure there is no mass on the system before turning off** the computer or the control box.
- Turn off the device control box.
- Shut down the computer so those without passwords cannot operate the device.
- Note: If there is a long time between testing participants, your computer settings may put the system to sleep and the wheels may lose power and drop. If you are leaving the computer for extended hours, shut down the system completely.

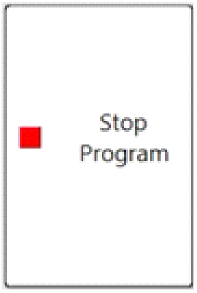


**Figure 2-17.** Stop program button

### 2-22 If You Stopped the Software and Want to Start Again (After Participant)

- If you tried to use the software and it did not respond, make sure that you put any buttons pressed back to their original status.
- Then, press the white arrow key in the upper left corner of the program – it should turn black to tell you the program is running.

*Note:*

- Never leave mass on the device unattended or after the device is powered down.
- Always turn the control box off after testing. It is not meant to be left on.

## 2-23 Troubleshooting

If the software program crashes, you probably pushed the wrong button at the wrong time.

- Inform the participant about the situation and ask them to lie still for the issue to be fixed.
- Never turn off the computer or the control box when the participant is being tested.
- Ask participant's permission to start over.
- Ask participants if they would like a rest before starting over. If so, remove all the weights, then remove the device from above the participant and make sure the participant is safely out of the device.
- You may have to stop the program and restart:
    - Click the small Stop Sign icon in the upper left of the program screen. **Figure 2-18**
    - The program is now stopped.
    - If the button you pressed is still pressed (it is not the usual color), press it to return it to the normal state.
    - Start the program again by clicking the Arrow Icon to the left of the Stop Sign Icon in the upper left screen. **Figure 2-18**
    - Re-do the problematic trial and resume the measurements.

- Depending on where you were in the program when it crashed, you might have to remove all the weights and go to the Participant Testing, Page 1 and return the wheels to the 0, 0, 0 position by clicking Return to 0, 0, 0. If the wheels do not return to the starting point for that participant, you will have to click the Kill XY Axes button and reposition the wheels to the Landing Site using the laser. You can then either teach new trajectories or use existing trajectories:
    - If using existing trajectories, skip the Teach Points section and just click the Set to 0, 0, 0, and Return to 0, 0, 0 buttons. Note, whenever you use existing trajectories, the participant must be exactly in the same position as when the trajectories were first collected.
    - If using new trajectories, you have to recollect all trials from 0N again.

- You may need to re-calibrate the device depending on the severity of the crash. In this case, you will have to recollect all trials.

- You may need to turn off the computer or control box as your last resort.

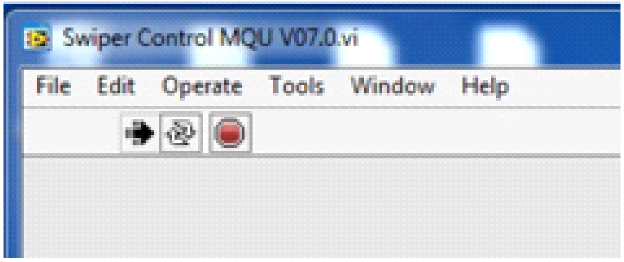


**Figure 2-18.** Program Start/Stop Buttons

# CHAPTER 3: Practical Considerations

Participants may be anxious about having weight placed on their back, as well as curious about the VerteTrack device and why spinal stiffness measurements are being taken. They also may not be familiar with anatomy and the rationale for recording stiffness data and so will not intuitively understand how their ability to follow instructions may affect data capture. Outcomes may be enhanced by performing some preliminary steps to familiarize the participant with clinical procedures. In this chapter, step-by-step instructions and examples are provided to improve the experience of both the operator and participant when using VerteTrack.

## 3-1 Participant Briefing (pre-test)

1. Briefly explain the rationale and goals for the spinal stiffness measurements and feedback training in terms appropriate to the participant's level of understanding.
2. Briefly explain the operation of the VerteTrack device
    a. Show participant the orientation video.
    b. Show the device to the participant in person, pointing out the different parts and explaining their function to help them further understand the process.
    c. Practice breathing protocols with the participant before beginning the measurements.
3. Explain to the participant what he or she can expect to feel and do during the session.

## 3-2 Example Remarks to Participant (pre-test)

"Low back pain is a major cause of pain, disability, and increased healthcare costs all over the world. Studies have shown that people with back pain seem to have more back stiffness. It has been also shown that with treatment, this stiffness may improve in some people but not others. We think that measuring spinal stiffness will help us to learn more about people with back pain and possibly teach us more about how we can help them. We can measure spine stiffness faster and more accurately using an instrument called a VerteTrack. This instrument includes a frame, a wheel system with a laser attached to it, and computer software.

We want you to be able to relax when we take the measurements so, please take your time to go to the restroom before we begin. During the test, you will lie face down and we may ask you to disrobe/change as necessary to expose your back (the test area) sufficiently. Gowns will be provided, but you will not need to change if you wear comfortable clothes that can be moved to expose your waistline. You will be also required to empty your front and back pockets of all objects and we ask that you remove your belt and glasses during the test. Once you are laying on the table, a researcher will touch your back in several places and then mark your skin with an ink pen. These marks are used to determine where we should measure the stiffness of your back. Next, to familiarize you with the instrument and the process we will perform a practice measurement.

During the test, you will feel the wheels roll over the skin of your back. This will feel a bit like a massage. There will be no needles, electric shocks, or other unexpected sensations. The only thing you should feel is the wheels touching your skin. We will add one light weight at a time to the machine which will simply make you feel like the wheels are adding more pressure to your back. This should not hurt at any time and most people are comfortable with having up to 6 weights placed on the machine. However, if you feel too much pressure, let us know and we will stop the measurements. During the test, we will guide you to hold an exhaled breath for about 10 seconds while data is recorded. We will let you know when you can return again to normal breathing. When we record the data, we ask that you do not move or talk to us, unless you need or want, to stop the test. Between measurements, we may ask you a few questions to ensure you are feeling well. When speaking to us, we ask that you do not move your head or body. We will be monitoring the results closely and if necessary, we may need to repeat one or more measurements to ensure high-quality data capture.

Remember that you can ask to stop immediately at any time if you feel you cannot or do not want to continue with your participation in the trial or in taking the measurements. You will not be penalized for doing so. Do you have any questions or concerns? …Shall we proceed?"

### 3-3 Participant Briefing (during the test)

1. Make sure that the participant follows all the instructions (breathing, movement, relaxation).
2. Monitor any participant reactions to testing and be sure to ask how participants feel when adding the weight plates.

### 3-4 Example Remarks to Participant (during the test)

"Relax your back and abdominal (stomach) muscles and try to remain still for the duration of the test (e.g.,15 min). We also ask that you try to keep your arms in the same position for the duration of the test because moving your arms can cause changes in the tone of muscles in your back which can affect measurements. Breathe normally unless instructed to hold your breath or exhale.

The wheels are on your skin now and we're going to start the test. Please take a deep breath in, now breathe out and hold it…hold…hold…keep holding…now you can breathe again. (Repeat with the participant through each addition of weight).

This is going to be the last measurement for now, but we ask that you not move or get off the table until we instruct you to. We will check the data and if everything looks good or if we need to repeat a measurement, we will let you know. Please wait for our instructions before you move away from the table"

### 3-5 Participant Briefing (post-test)

1. Make sure that the participant gets up safely.
2. Explain to the participant what he or she can expect to feel and do after the session.

### 3-6 Example Remarks to Participant (post-test)

After checking all the graphs: "We are done with our measurements. When it is time, I will help you sit up from the plinth."

After removing the device assist the participant while saying: “Slowly get up from the table. If you need assistance, let us know. You may experience some dizziness; if so, please advise us of it and stay seated for a few minutes before standing up.”

Discharging the participant: “You may feel slight discomfort or back stiffness or show signs of mild skin irritation following our examination today. These things are normal and any discomfort you feel should improve on its own within two days, however, if it does not subside or you feel worried do not hesitate to contact the principal investigator.”

## 3-7 Participant Briefing (between tests)

1. Depending on what is being investigated, you may need to control for different activities including exercise, food intake, hydration levels (e.g., abdominal contents, gas, delayed onset muscle soreness, etc.).
2. Ask participants to refrain from treatments to the spine between sessions unless this treatment is a part of the experiment being conducted.
3. If the two testing sessions are a few hours apart, ask the participant to go for a walk and remind them to use the restroom before the second session starts.

## 3-8 Example Remarks to Participant (between tests)

“During the time between your test sessions, try to avoid any activities that could change the strength of your back muscles, for example, heavy weightlifting or personal training and performing sit-ups or other core strengthening exercises). You may continue with your usual activities (at home and at work) but we ask that you do not start any new exercises between your visits to our lab. If you take medication like muscle relaxants or pain killers, try to take the medication after the assessment.”

## 3-9 Recommendations for Operators to Reach the Same Position over Multiple Measurement Sessions

- Use a permanent marker (particularly for S1) to ensure the starting position for each measurement is the same.

- Ask the participant not to remove the ink marks on purpose or to add other marks to the test area.
- Use a standardized examination plinth with armrests and a face hole for prone positioning.
- Take a photo of the markings on the back (if allowed in your ethics procedure) to aid in repositioning the wheels on future testing dates.
- Consider using a band-aid/ adhesive tape to cover the marked "x" spot so you don't lose it for the next visit.
- Measure the trajectory distance.

## 3-10 Summary of Recommendations for Operators to Optimize Participant Safety

- Familiarize yourself with the location of the hardware emergency stop (E-stop) before performing patient assessment using the VerteTrack.
- The safety stop button should immediately elevate the load and return the rolling arm to a position away from the participant to allow them to exit the device if needed.
- Before participant testing **only**, check the E-stop by depressing the emergency stop and then disengage it to ensure it is working.
- Ensure participants know that they can request the testing b stopped at any time and without penalty.
- Use an easy reading instruction format for clients with disabilities before assessment by VerteTrack.
- Disinfect the wheels/bench/equipment prior to seeing each participant.
- Provide clear instructions to participants with expectations explained.
- Follow the suggested pre-test protocol to make sure all "detectors" are functioning properly.
- Make sure the device is properly operational (or locked in place) when loading weights.
- Continue to check in with the participant throughout the process to make sure that they are feeling okay.
- Make sure all of the weights are removed from the device before and after assessment by the VerteTrack.
- Make sure to remove the weights one by one at the end of the measurement.

- Do not allow the participant to get up before the frame has been moved away from them.
